# Aminooxy acetic acid suppresses Th17-mediated psoriasis-like skin inflammation by inhibiting serine metabolism

**DOI:** 10.3389/fphar.2023.1215861

**Published:** 2023-08-15

**Authors:** Jong Yeong Lee, Ji-Hyun Lee, Hyo Jung Lim, Eonho Kim, Dae-Ki Kim, Jin Kyeong Choi

**Affiliations:** ^1^ Department of Immunology, Jeonbuk National University Medical School, Jeonju-si, Republic of Korea; ^2^ Department of Physical Education, Dongguk University, Seoul, Republic of Korea

**Keywords:** psoriasis, aminooxy acetic acid, serine metabolism, Th17, Treg, mTOR

## Abstract

**Background:** Psoriasis is a common chronic inflammatory skin disease characterized by an external red rash that is caused by abnormal proliferation and differentiation of keratinocytes and immune T cells. This study aimed to elucidate the role of aminooxy acetic acid (AOA) in alleviating psoriasis from the perspective of immunology and metabolomics. Therefore, contributing to the development of new drugs as candidates for psoriasis treatment.

**Methods:** To investigate the symptom-alleviating effects and the related mechanisms of AOA on the treatment of psoriasis, we used a 12-O-tetradecanoylphorbol-13-acetate-induced psoriasis-like skin mouse model and interleukin (IL)-17-stimulated human keratinocytes.

**Results:** The results showed that AOA ameliorated psoriasis-related symptoms and decreased inflammation-associated antimicrobial peptides and T-helper 17 (Th17)-associated cytokines in a mouse model of psoriasis. Furthermore, AOA inhibited the activation of mechanistic target of rapamycin (mTOR) by suppressing serine metabolism-related genes. Importantly, mTOR inhibition ameliorated psoriatic disease by affecting the differentiation of various T cells and normalizing the Th17/regulatory T (Treg) cell balance. In addition, IL-17-stimulated human keratinocytes showed the same results as in the *in vivo* experiments.

**Conclusion:** Taken together, these results suggest that targeting the serine metabolism pathway in the treatment of psoriasis is a novel strategy, and that AOA could be utilized as a novel biologic to treat psoriasis.

## 1 Introduction

Psoriasis has an estimated global prevalence of 3%–4% and is a chronic recurrent inflammatory immune-mediated skin disease (Michalek et al., 2017). Psoriasis is characterized by clinical symptoms such as distinct red and scaly plaques, and histological symptoms such as hyperproliferation of keratinocytes, dermal inflammatory cell infiltration, and angiogenesis (Pasparakis et al., 2014). In psoriasis, the eruptions appear mainly on the elbows and knees initially, and small eruptions on the skin gradually spread, covering the skin of the whole body with the eruption. In addition to skin eruptions, psoriasis is associated with psoriatic arthritis, autoimmune disease, cardiovascular disease, and metabolic disease. Moreover, the risk of cancer is higher in patients with psoriasis (Furue et al., 2020). The pathogenesis of psoriasis remains unclear; however, it is mainly associated with an abnormal immune response caused by the stimulation of genetic and environmental factors (Kamiya et al., 2019).

Because of the complex cellular and related molecular pathogenesis of psoriasis, no satisfactory strategies have been developed to treat this disease. Currently used drugs for psoriasis include methotrexate, cyclosporine, the phosphodiesterase 4 inhibitor apremilast, ixekizumab, dithranol, guselkumab, brodalumab, and secukinumab (de Carvalho et al., 2017). However, the use of these drugs has many adverse effects that limit their clinical application. These include drug resistance, hormonal imbalance, high recurrence rate, and poor prognosis (Jiang et al., 2020). In addition, treatments such as mild topical therapy, phototherapy, steroids, and vitamin D3 analogs suppress psoriasis inflammation, but these have less coverage and are less effective (Mason et al., 2002). Therefore, the development of novel safe and effective anti-psoriatic drugs is urgently required.

Aminooxy acetic acid (AOA) suppresses pyridoxal-5′-phosphate-dependent transaminases, which regulate the interconversion of alpha (α)-amino and α-keto acids. The redox balance of this reaction is maintained by converting the nitrogen donor, glutamate, to α-ketoglutaric acid (Son et al., 2013). According to a previous study, AOA attenuated autoimmune encephalomyelitis and suppressed the severity of autoimmune uveitis by modulating the fate of T cells (Wang et al., 2016; Mei et al., 2020). Thus, AOA affects T cell differentiation by regulating amino acid metabolic pathway-related genes.

Psoriasis is caused by hyper immunity, which causes abnormalities in immune function. In psoriasis, T-cell activity increases causing excessive secretion of immune substances that induce keratinocyte proliferation and inflammation (Hu et al., 2021). Both T helper type 17 (Th17) and regulatory T (Treg) cells are increased in psoriasis skin lesions. Th17 and Treg cells play critical roles in the immune response and antagonize each other in immune disorders. Therefore, the Th17/Treg balance is important for maintaining immune homeostasis in the body (Zhang et al., 2021). However, the proportion of Treg cells in psoriatic skin tissue is relatively low, which leads to a hyperimmune response. As such, psoriasis is caused by an imbalance of Th17 and Treg cells. Therefore, the solution to this is to increase the number of Treg cells to rescue the balance between them.

Skin immune cells can engage in metabolic pathways of certain nutrients to maintain homeostasis, and high levels of amino acids and metabolites can promote or control the recurrence of skin diseases (Cibrian et al., 2020). Serine is a non-essential amino acid that can be taken directly into cells or synthesized via the serine biosynthetic pathway by 3-phosphoglycerate (3-PG), an intermediate in the glycolysis pathway (Zeng et al., 2019). Serine has recently been reported as an essential metabolite for T cell and keratinocyte proliferation (Ma et al., 2017; Cappello et al., 2022). In addition, T cell, keratinocyte proliferation and other inflammatory cell expansion were reduced without exogenous serine/glycine (Ma et al., 2017; Cappello et al., 2022). Based on these reports, limiting serine metabolism may have substantial therapeutic implication for immunotherapy and inflammatory immune cell regulation in diseases. Therefore, we investigate the regulate the pathogenesis of Th17-mediated psoriasis-like skin by inhibiting the serine metabolism using AOA.

## 2 Material and methods

### 2.1 Animals

Nine-week-old female C57BL/6J mice (*n* = 20) were purchased from NARA Biotech (Pyeongtaek, Korea) and housed in a laminar cabinet. Throughout this study, mice were maintained at 22 ± 2°C, 55% ± 5% humidity, and in a 12 h light/dark cycle. The research protocol was approved by the Institute Animal Protection and Utilization Committee of Jeonbuk National University (approval number JBNU 2021-0169).

### 2.2 TPA induced psoriasis model

TPA was diluted in acetone and dimethyl sulfoxide (DMSO) (7:1) (Hiraganahalli Bhaskarmurthy and Evan Prince, 2021). Both ear surfaces were treated from day zero to seven (2.5 μg/mouse). The mice used in this experiment was assigned to four groups (*n* = 5 per group) as follows: control, TPA, TPA plus AOA (125 and 250 μg/mouse). AOA was administered seven times to the abdominal cavity. During the same period, non-immune control mice and TPA-induced psoriasis mice were administered phosphate-buffered saline (PBS) to the abdominal cavity. During the 7 days of the experimental period, ear thickness was evaluated with a dial thickness measuring device (Mitutoyo Co., Tokyo, Japan) as an inflammatory indicator, and the weight of the mouse was measured. After the final treatment, the mice were euthanized using CO_2_ and ear samples were acquired and used for further analysis. Part of the ear tissue was stored in a −80°C freezer for subsequent RNA and protein analysis, while another ear tissue was fixed in 4% formaldehyde for pathological analysis. Furthermore, to isolate single cells from the ear skin, the skin tissue was placed in a fresh enzyme medium (RPMI-1640 medium containing 0.4 mg/ml Liberase from Roche and penicillin/streptavidin) at 37°C for 1.5 h. Subsequently, the cells were disrupted and filtered using a 70 μm cell strainer. The filtered single cells were then analyzed by flow cytometry.

### 2.3 Flow cytometry

On day seven, the ears of TPA-induced psoriasis mice were collected and finely ground. The cells were then soaked in media containing Liberase (Roche) and incubated for 1 h and 30 min. The cells were filtered using a 70 μm Nylon cell filter, centrifuged at 400 × g for 8 min, and red blood cells were removed with ammonium-Chloride-Potassium (ACK) lysing buffer and washed with PBS (Lorenz and von Stebut, 2014). The separated cells were stimulated for 4 h using phorbol-12-myristate-acetate (PMA), ionomycin, and a Golgi plug kit (BD Pharmingen). A viability staining kit (Invitrogen) was used to distinguish dead cells. Tregs and cytokines were expressed as anti-mouse CD4 (clone RM4-5, Biolegend), anti-mouse IFN-γ (clone XMG1.2, BD Pharmagen), anti-mouse Foxp3 (clone FJK-16s, eBioscience), anti-mouse IL-10 (clone JES5-16E3, BD Pharmagen), anti-mouse IL-17A (clone TC11-18H10, BD Pharmagen). In the FACS analysis, cells were stained with fluorescent dyes and monoclonal antibodies, and dead cells were excluded. Each cell was classified according to the fluorophore of the antibody, and the gate in the analysis was set using less than 0.5% isotype control. The FACS analysis was performed using a software from the manufacturer Attune NxT Flow Cytometer (Invitrogen).

### 2.4 Histological analysis

The ears (*n* = 20) were fixed with 4% formaldehyde and placed in paraffin. The sections were cut at 5 μm and stained with hematoxylin and eosin (H&E). Skin thickness was measured by H&E staining in five randomly selected fields at ×200 magnification for each sample.

### 2.5 Cell culture and cell viability analysis

The HaCaT human keratinocyte cell line was purchased from CLS Cell Lines Service (Eppelheim, Germany). Cells were cultured in 10% fetal bovine serum (FBS), Dulbecco’s Modified Eagle Medium (DMEM) containing antibiotics (100 U/mL penicillin G, 100 μg/mL streptomycin), 5% CO_2_, and 37°C.

IL-17A (100 ng/ml; PeproTech) Serine (400 μM; Sigma), glycine (400 μM; Sigma), and SHMT1/2 inhibitor RZ-2994 (2 μM; Cayman Chemical) were added to cell culture media as indicated. Cell viability was measured by 3-(4,5-dimethylthiazol-2-yl)-2,5-diphenyltetrazolium bromide (MTT) analysis. AOA was added at different concentrations for 24 h. Subsequently, MTT (5 mg/mL) was added to each well, and the plate was incubated for 2 h. Formazan crystals were dissolved by treatment with DMSO, and the absorbance of each sample was expressed as a percentage of the control group.

### 2.6 ELISA

HaCaT cells were activated *in vitro* for 48 h by stimulation with IL-17A (100 ng/ml) in presence or absence of AOA or RZ2994. Supernatants were collected after a 48 h in culture. Serine levels were quantified using kits from Serine ELISA kit (Abcam).

### 2.7 Quantitative real time polymerase chain reaction (qPCR)

qPCR was performed to confirm the mRNA expression in the ears of HaCaT cells and mice, and RNA was isolated using RNAiso Plus (Takara Bio). Reversal transfer was performed at 45°C (60 min) and 95°C (5 min). cDNA (100 ng) were mixed with 1 μL (0.4 μM) of forward and reverse primer solutions. The final mixture (10 μL) was obtained using 5 μL of SYBR premix (Nanohelix, Daejeon, Korea) and dH_2_O (1 μL). The primers used are listed in Supplementary Table S1. Normalization and quantification of mRNA expression were performed using the StepOnePlus Real-Time PCR (Applied Biosystems) software.

### 2.8 Western blot

On day seven of the experiment, the ears and cells of TPA-induced psoriasis mice were homogenized and dissolved, and western blotting was performed. After mixing Tissue Protein Extraction Reagent (T-PER), Phosphor stop, and ethylenediaminetetraacetic acid (EDTA)-free, 200 μL was added to each ear for homogenization, pulverization, and centrifugation at 10,000 × g for 5 min to use only the supernatant. In the case of cells, IL-17A (100 ng/mL) stimulated (1×10^6^ cells/well in a six-well plate) the cells for 48 h to induce the production of signaling molecules. Cells were washed with cold PBS, 100 μL of lysate buffer was added, and cells were sonicated for 30 s. Subsequently, centrifugation was performed for 10 min to collect the supernatant. The protein extract (20 μg/lane) was fractionated by 6%–10% gradient sodium dodecyl sulfate-polyacrylamide gel electrophoresis (SDS-PAGE) and then transferred to a polyvinylidene fluoride (PVDF) membrane (Bio-Rad). A tris-buffered saline Tween 20 (TBS-T) buffer containing 5% bovine serum albumin (BSA) was lightly stirred at room temperature for 1 h and then treated with a specific antibody solution at 4°C overnight. The antibodies used were to detect phosphoglycerate dehydrogenase (PHGDH) (Abcam), phosphohydroxythreonine aminotransferase (PSAT)1, phosphoserine phosphatase (PSPH) (Invitrogen), phosphorylated (p)-AMP-activated protein kinase (AMPK)α, AMPKα, p-mTOR, mTOR, serine hydroxy methyltransferase (SHMT)1, SHMT2, and β-actin (Cell Signaling Technology). The PVDF membrane was washed with TBST buffer to remove redundant antibody structures, and the secondary antibody was stirred gently at room temperature for 1 h. Immunodetection was performed using Super Signal™ West Pico PLUS Thermo Scientific.

### 2.9 Th17 cell polarization

Naïve CD4^+^ T cells were isolated from the total splenocytes of 6-weeks-old C57BL6/J mice using the CD4^+^CD62L^+^ T cell isolation kit (Miltenyi Biotec) according to manufacturer’s protocol. The isolated naïve CD4^+^ T cells were then polarized into Th17 cells by adding 25 ng/ml IL-6 (R&D systems), 3 ng/ml TGF-β (PeproTech), 10 μg/ml anti-IL-4 (R&D systems), and 10 μg/ml anti-IFN-γ (R&D systems) in presence of plate bound anti-CD3 (5 μg/ml, BioXcell) and anti-CD28 (5 μg/ml, BioXcell) for 5 days. The cells were cultured in TexMACS Medium (Miltenyi Biotec) with 10% FBS, 1% penicillin/streptomycin and 1% glutamate (Sigma-Aldrich). The treatment of AOA on Th17 cells was conducted on day 3 and analyzed on day 5.

### 2.10 Statistical analysis

GraphPad Prism 9 was used for data analysis, and one-way analysis of variance (ANOVA) or *t*-test was used, as well as the Holm-Šídák *post hoc* test. Statistical significance was set at *p* < 0.05, and the data are expressed as mean ± standard error of the mean (SEM).

## 3 Results

### 3.1 AOA ameliorates skin pathology and inhibits amplified psoriasis-associated genes in mouse model

**FIGURE 1:**
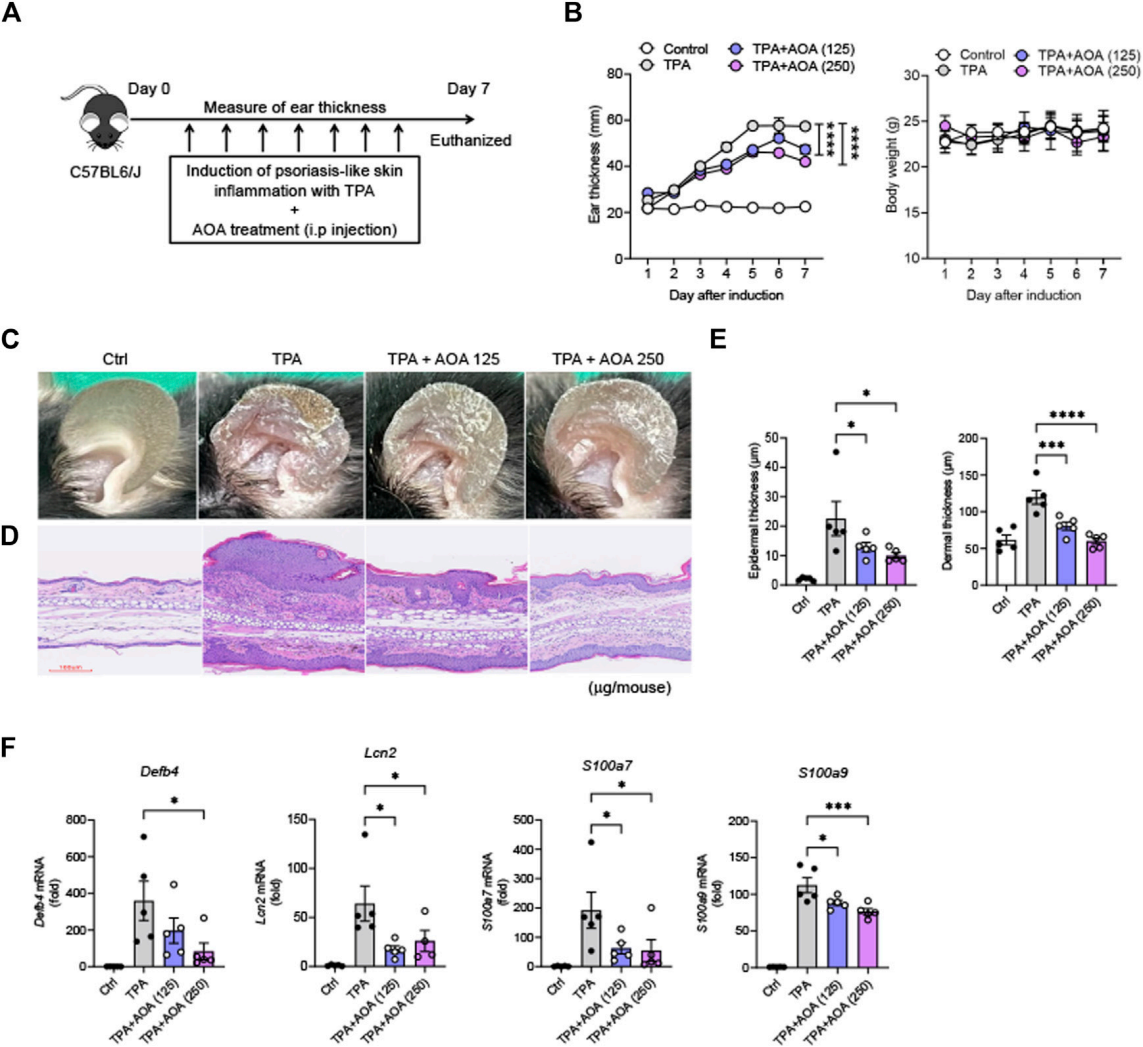
AOA confers protection against TPA-induced psoriasis mouse model. **(A)** Schematics showing the experiment schedule for the animal model of TPA-induced psoriasis and treatment strategy. **(B)** Ear thickness (mm) and body weight change (g). **(C)** Representative photographs of mouse ear skin in each group. **(D)** Representative images of H&E staining (×200 magnification; scale bar = 100 μm) in each group. **(E)** Epidermal and dermal thickness (μm). **(F)** The mRNA expression of psoriasis-associated antimicrobial peptides (*Defb4*, *Lcn2*, *S100a7*, and *S100a9*). All data are showed as the mean ± SEM of three independent experiments. Values were analyzed by Holm-Šídák *post hoc* test. *p < 0.05, **p < 0.01, ***p < 0.001, and ****p < 0.0001 vs TPA-induced only group. AOA, aminooxy acetic acid; TPA, 12-O-tetradecanoylphorbol-13-acetate; H&E, hematoxylin and eosin; SEM, standard error of the mean; Defb4, defensin β 4; Lcn2, lipocalin 2.

We derived an experimental animal model of psoriasis to investigate the alleviating effects of AOA and related mechanisms of psoriasis symptoms. AOA was injected daily intraperitoneally (i.p.) (125 μg/mouse or 250 μg/mouse; i.p.) for 7 days (Figure 1A). In this study, mice in the group in which psoriasis-like skin was induced by TPA showed significant thickening of the ear skin compared to mice in the normal group. However, AOA attenuated ear skin thickening during psoriasis progression in mice. Compared to the normal group, no significant difference in body weight was observed between the TPA and AOA treatment groups (Figure 1B). In addition, we visually confirmed that the administration of AOA caused a significant recovery of psoriasis-like symptoms in the ear skin of the TPA-induced psoriasis model (Figure 1C). H&E staining was performed to analyze histopathological changes such as epidermal hyperkeratosis and hypertrophy in the ear tissues of each group. The epidermal and dermal thicknesses were significantly greater in the TPA group than in the normal group. However, AOA treatment alleviated the TPA-induced epidermal and dermal thickening of ear tissues (Figures 1D, E). To examine the anti-inflammatory effect of AOA on psoriasis, we observed changes in the expression of antimicrobial peptides, such as defensin β 4 (*Defb4*), lipocalin 2 (*Lcn2*), *S100a7*, and *S100a9,* which are known to amplify local inflammatory processes. As shown in Figure 1F, the mRNA expression levels of antimicrobial peptides increased by TPA were significantly decreased after treatment with AOA.

### 3.2 AOA inhibits Th17 and induces Treg cells during psoriasis-like skin inflammation

**FIGURE 2:**
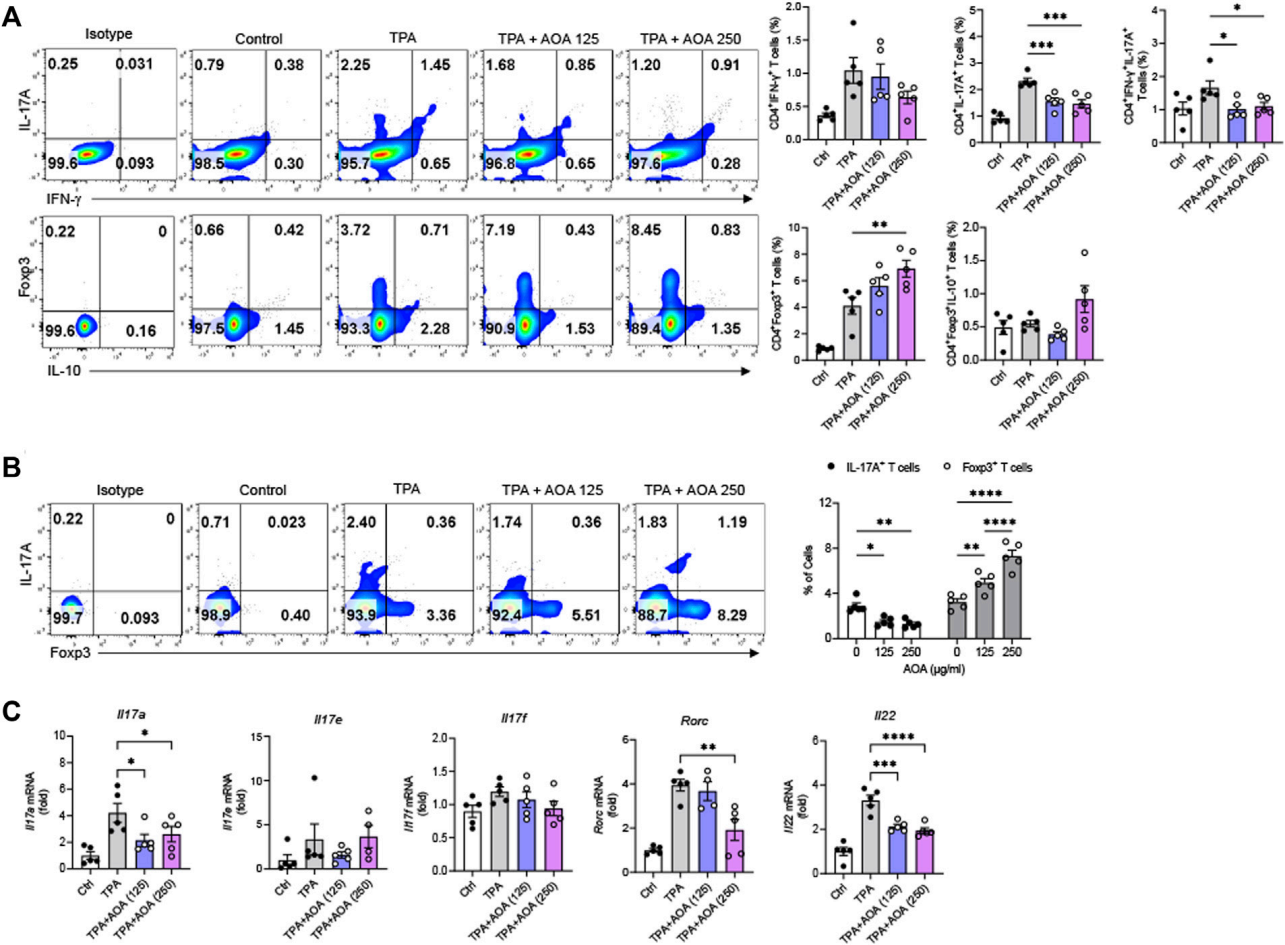
AOA reprograms Th17 cell expansion into Treg cells in the TPA-induced psoriatic skin. Cells of the ear skin were collected from each mouse and analyzed by FACS. **(A)** Analysis of changes in IFN-γ-, -IL-17A-, Foxp3-, or IL-10-expressing CD4^+^ T cells in mouse ear skin cells by AOA treatment in TPA-induced psoriasis mice. **(B)** AOA reprograms Th17 cell differentiation into Treg cells. **(C)** The mRNA expression of psoriasis-associated Th17 cytokines (*IL17A, IL17E, IL17F, RORC, and IL22*). All data are showed as the mean ± SEM of three independent experiments. Values were analyzed by Holm-Šídák *post hoc* test. *p < 0.05, **p < 0.01, ***p < 0.001, and ****p < 0.0001 vs TPA-induced only group. AOA, aminooxy acetic acid; TPA, 12-O-tetradecanoylphorbol-13-acetate; H&E, hematoxylin and eosin; SEM, standard error of the mean; Th17, T helper type 17; IL, interleukin; FACS, fluorescent activated cell sorting; RORC, RAR related orphan receptor C.

During the inflammatory process of psoriasis, naïve CD4^+^ T cells differentiate into Th1 or Th17 cells upon stimulation with various cytokines, promoting the activity of the adaptive immune system (Hiraganahalli Bhaskarmurthy and Evan Prince, 2021). In addition, immune dysfunction occurs due to an imbalance of T cells, in which the proportion of Treg cells that regulate immune excess is relatively low compared to Th17 cells in psoriasis (Zhang et al., 2021). Therefore, to investigate how AOA affects the regulation of T cells in skin inflammatory lesions in a TPA-induced psoriasis-like mouse model, we isolated cells from the ear and identified them using FACS. As expected, the number of Th17 cells in the ear of the AOA-treated mice was significantly reduced compared to that in the untreated group, and the number of Treg cells increased. The number of Th1 cells was decreased by AOA treatment compared with that in the TPA group, but the difference was not significant. In addition, there was no increase in the number of Foxp3^+^IL-10^+^ IL-10-expressing Tregs (Figure 2A). AOA significantly reduced the expansion of IL-17A-producing Th17 cells and significantly induced the expansion of Foxp3^+^ Treg cells in psoriasis-like skin (Figure 2B). This suggests that AOA has a reprogramming function that inhibits Th17 cell differentiation and promotes Treg cell differentiation in a dose-dependent manner. In addition, AOA treatment significantly decreased the expression levels of *Il17a,* RAR related orphan receptor C (*Rorc*), and *IL22* in ear skin of the psoriasis-like mouse model. *Il17e* and *Il17f* also decreased compared to the TPA group, but the difference was not significant (Figure 2C).

### 3.3 AOA inhibits mTOR via serine metabolism in psoriasis-like skin inflammation

**FIGURE 3:**
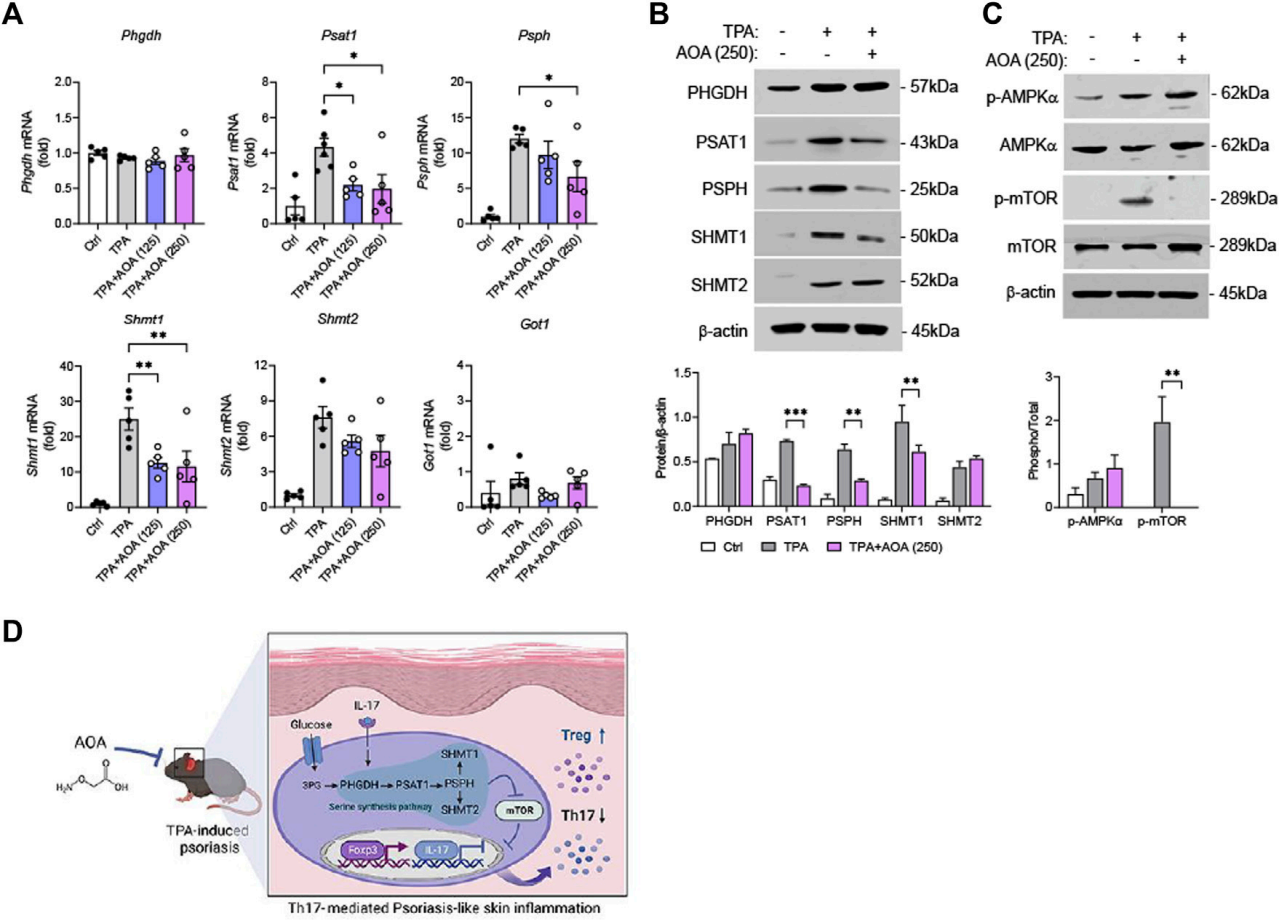
AOA inhibits serine metabolism and mTOR pathway in the TPA-induced psoriatic skin. **(A)** The mRNA expression of serine metabolism related genes of mouse ear tissues analyzed by qPCR. **(B)** The protein expression of serine metabolism related genes of mice ear tissues as analyzed by western blot, and bar graph of the relative intensities. **(C)** The protein expression of AMPK/mTOR of mice ear tissues as analyzed by western blot, and bar graph of the relative intensities. All data are showed as the mean ± SEM of three independent experiments. **(D)** A schematic diagram of the effects of AOA in the TPA-induced psoriasis-like model. AOA inhibits the serine metabolism-mTOR axis pathway activated in mouse skin tissue and subsequently reduces Th17-mediated inflammatory cytokines. Values were analyzed by Holm-Šídák *post hoc* test. *p < 0.05, **p < 0.01, ***p < 0.001, and ****p < 0.0001 vs TPA-induced only group. AOA, aminooxy acetic acid; TPA, 12-O-tetradecanoylphorbol-13-acetate; H&E, hematoxylin and eosin; SEM, standard error of the mean; qPCR, quantitative real time polymerase chain reaction; mTOR; mechanistic target of rapamycin; AMPK, AMP-activated protein kinase.

We investigated the underlying mechanisms to determine through which pathway AOA reprograms T-cell differentiation and alleviates the symptoms of psoriasis. Previous studies have shown that AOA is a pan-transaminase inhibitor that inhibits the synthesis of several amino acids (aspartate, glycine, alanine, and serine) (Xu et al., 2017). Therefore, we investigated the effect of AOA on the expression of serine metabolic components in TPA-induced psoriasis-like skin lesions. First, we verified the expression levels of the serine biosynthesis pathway (*Phgdh*, *Psat1* and *Psph*) and serine entry into the one-carbon metabolism pathway (*Shmt1* and *Shmt2*) using qPCR. As shown in Figure 3A, AOA significantly inhibited the expression of *Psat1* and *Psph* without affecting the expression of *Phgdh* in the serine biosynthesis pathway. In addition, the expression of *Shmt1* in serine entry into the one-carbon metabolism pathway was significantly suppressed by AOA. The expression of *Shmt2* was also decreased by AOA, but the difference was not statistically significant. Notably, AOA did not significantly affect the expression of glutamic-oxaloacetic transaminase 1 (Got1), indicating that treatment with AOA did not affect aspartic biosynthesis in the psoriatic skin environment. Furthermore, protein expression was confirmed by western blotting. The expression of *Psat1*, *Psph*, and *Shmt1* was significantly reduced by AOA in TPA-induced psoriatic skin, consistent with the qPCR results (Figure 3B). We next investigated the protein expression changes in AMPK activation and mTOR phosphorylation by AOA in TPA-induced psoriatic skin. The results showed that AOA treatment attenuated the phosphorylation of mTOR without significantly affecting AMPK activity (Figure 3C). Taken together, AOA can reduce the production of Th17-mediated inflammatory factors and increase Foxp3^+^ Tregs by inhibiting both activated serine metabolism and mTOR in the skin of a TPA-induced psoriasis mouse model (Figure 3D).

### 3.4 Immuno-suppressive effects of AOA require serine metabolism in IL-17-mediated psoriatic keratinocytes

**FIGURE 4:**
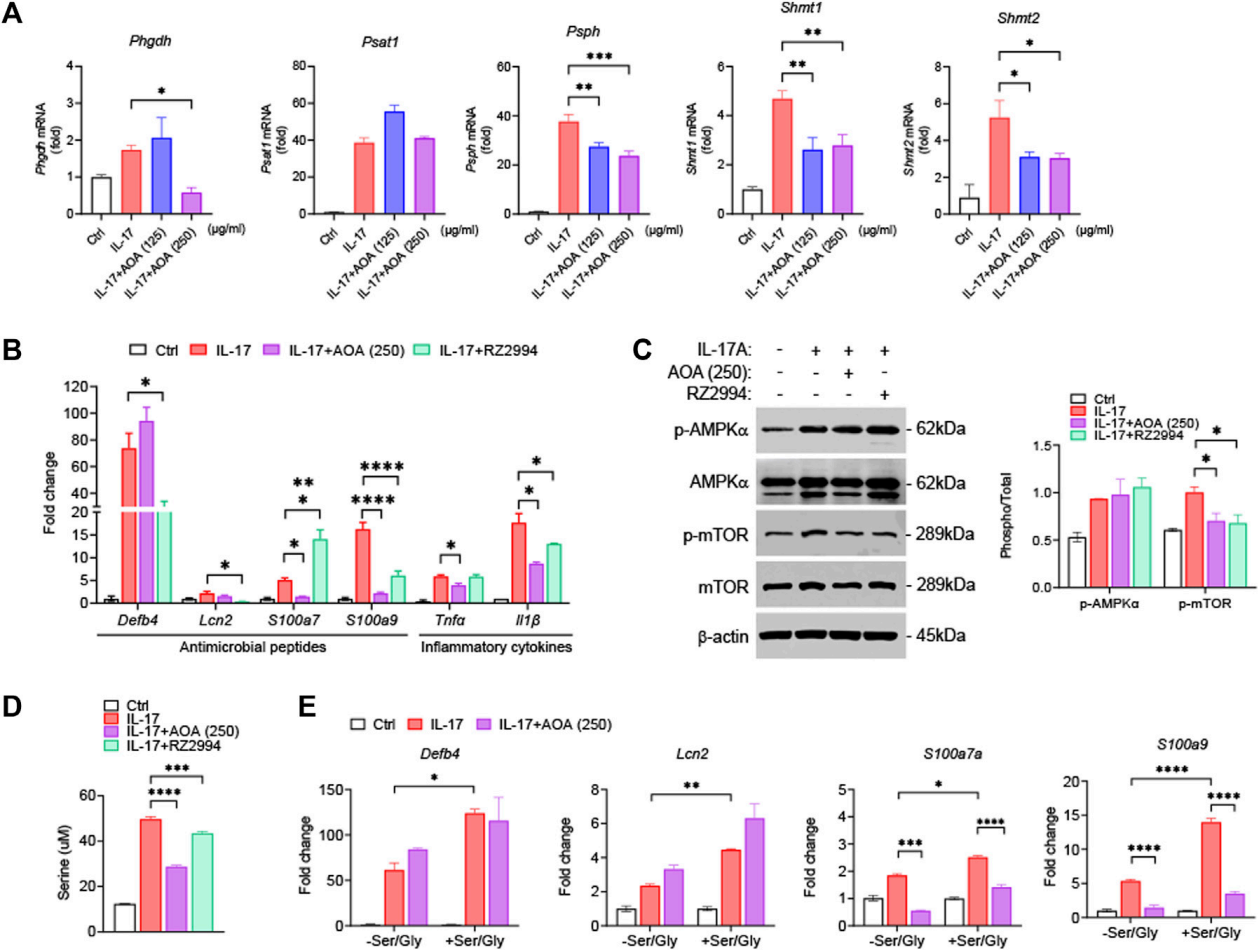
AOA ameliorates IL-17-mediated keratinocyte inflammation by inhibiting mTOR through the restriction of serine metabolism. **(A)** The mRNA expression of serine metabolism related genes of human keratinocytes analyzed by qPCR. **(B)** The mRNA expression of psoriasis-associated antimicrobial peptides (*Defb4, Lcn2, S100a7*, and *S100a9*) by human keratinocytes analyzed by qPCR. **(C)** The protein expression of AMPK/mTOR by human keratinocytes analyzed by western blot, and bar graph of the relative intensities. **(D)** Analysis of serine levels from the supernatants were analyzed by ELISA. **(E)** Gene expression of IL-17A-stimulated human keratinocytes cultured in serine/glycine-free medium, or serine/glycine-free medium containing serine (400 μM) and glycine (400 μM) in the presence or absence of AOA. All data are showed as the mean ± SEM of three independent experiments. Values were analyzed by Holm-Šídák *post hoc* test. *p < 0.05, **p < 0.01, ***p < 0.001, and ****p < 0.0001 vs IL-17-stimulated only group. AOA, aminooxy acetic acid; SEM, standard error of the mean; qPCR, quantitative real time polymerase chain reaction; mTOR; mechanistic target of rapamycin; AMPK, AMP-activated protein kinase; Defb4, defensin β 4; Lcn2, lipocalin 2.

To examine whether the effect of AOA on the inhibition of psoriasis-related mechanisms *in vitro* was similar to those *in vivo*, we used IL-17-stimulated HaCaT cells. First, we investigated the effect of AOA on the viability of HaCaT cells. We confirmed that AOA treatment at concentrations of 0–250 μg/mL did not induce cytotoxicity in HaCaT cells (Supplementary Figure S1). Certain metabolic pathways control cell activation/proliferation/differentiation and modulate the shift towards inflammatory or anti-inflammatory responses. In psoriasis, immune-metabolic interactions may orchestrate IL-17-induced pathogenicity, which can induce skin plaques as well as systemic inflammatory responses and psoriasis-related comorbidities (Krueger and Brunner, 2018). Further, IL-17-mediated keratinocyte psoriasis is associated with increased levels of various inflammatory cytokines in the skin. Recent findings have highlighted serine/glycine control as a metabolic hub that can regulate the proliferation and expansion of inflammatory cells (Cappello et al., 2022). Therefore, we examined the whether the effects of AOA on the expression of serine metabolism-related genes in IL-17-stimulated HaCaT cells. As shown in Figure 4A, all genes involved in serine metabolism were significantly increased in IL-17-stimulated cells compared to those in non-stimulated control cells. The expression of *Phgdh* was significantly decreased by treatment with a high concentration of AOA, and no significant change in *Psat1* was observed after AOA treatment. AOA showed a significant inhibitory effect on the expression of *Psph, Shmt1*, and *Shmt2* at all the tested concentrations. To compare and observe the anti-inflammatory effect of AOA and RZ2994, a known SHMT1/SHMT2 inhibitor, in IL-17-stimulated HaCaT cells, we examined the changes in the expression of antimicrobial peptides (*Defb4, Lcn2, S100a7,* and *S100a9*) and inflammatory cytokines (*Tnfα* and *Il1β*). As a result, the expression levels of *Lcn2, S100a7, S100a9, Tnfα,* and *Il1β*, which were increased by IL-17 stimulation, were found to be decreased upon AOA treatment. Additionally, the expression of *Defb4*, *Lcn2*, *S100a9*, and *Il1β*, which elevated after stimulation with IL-17 were reduced by RZ2994 (Figure 4B).

We next investigated whether AOA or RZ2994 could inhibit mTOR phosphorylation by activating AMPK in IL-17-stimulated HaCaT cells. Both AOA and RZ2994 significantly inhibited mTOR phosphorylation. However, neither AOA nor RZ2994 increased AMPK activation (Figure 4C). These results, consistent with *in vivo* experiments, suggest that AOA can inhibit inflammatory mediators by directly regulating mTOR phosphorylation without requiring AMPK activation by inhibiting serine metabolism.

Next, to clarify the anti-inflammatory effect of AOA through the serine metabolic pathway in psoriasis-like skin inflammation, we investigated whether AOA regulates the extracellular released serine levels by inhibiting intracellular serine synthesis. AOA inhibited expression of intracellular serine synthesis enzymes and demonstrated a significant downregulation of extracellular released serine levels, as estimated by ELISA (Figure 4D). Additionally, to understand the role of extracellular serine utilization in keratinocytes, we added serine/glycine (glycine was added because serine can be reversibly converted to glycine by SHMT) to IL-17A-stimulated keratinocytes. We observed a more significant increase in expression of IL-17-mediated inflammatory genes in keratinocytes supplemented with serine/glycine than in those lacking serine/glycine (Figure 4E). Taken together, these results indicated that the inflammatory response of keratinocytes mediated by IL-17 depends on the supply of serine, and AOA reduces the inflammatory response of keratinocytes through the restriction of serine metabolism.

### 3.5 AOA suppresses serine metabolism in Th17 cells

**FIGURE 5:**
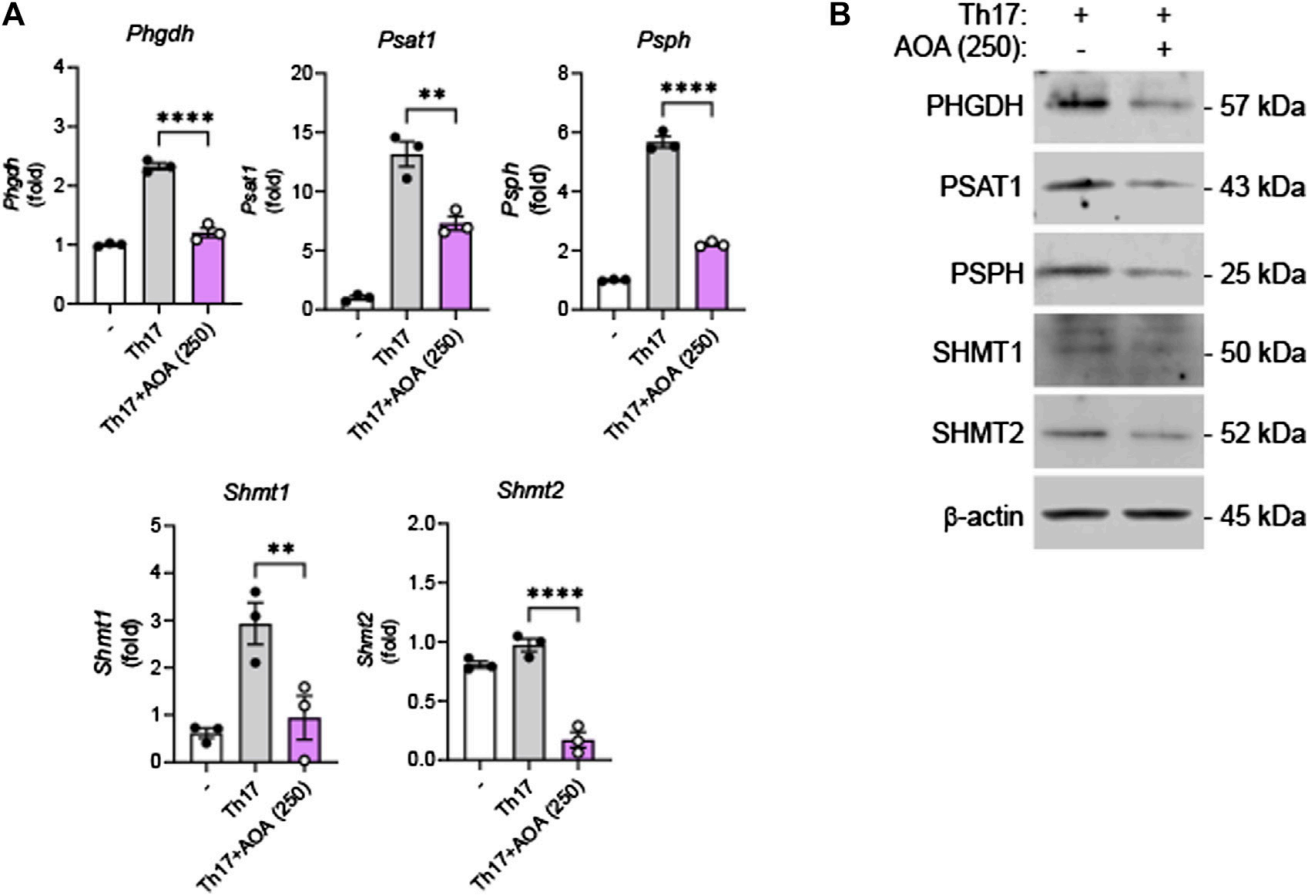
Inhibition of serine metabolism in Th17 cells by AOA. Splenic CD4^+^CD62L^+^ naïve T cells from C57BL6/J mice were isolated and polarized under Th17 conditions with anti-CD3/28 antibodies for 5 days. **(A)** The mRNA expression of serine metabolism related genes of mouse primary Th17 cells analyzed by qPCR. **(B)** The protein expression of serine metabolic pathways by mouse primary Th17 cells analyzed by western blot, and bar graph of the relative intensities. All data are showed as the mean ± SEM of three independent experiments. Values were analyzed by Holm-Šídák *post hoc* test. *p < 0.05, **p < 0.01, and ****p < 0.0001.

It has been reported that effector T cells, which proliferate rapidly *in vitro* and *in vivo*, require a sufficient supply of serine, and serine restriction can inhibit T cell expansion (Ma et al., 2017). After demonstrating that AOA reduces inflammation by inhibiting serine metabolism in Th17-mediated psoriasis, we investigated whether this drug could also reduce the inhibition of serine metabolism and IL-17A production in mouse primary Th17 cells. Consistent with the *in vivo* results, AOA inhibited IL-17A production in Th17 cells (Supplementary Figure S2). Furthermore, both RNA and protein levels of serine metabolic enzymes (PHGDH, PSAT1, PSPH, and SHMT1/2) were considerably reduced in AOA-treated Th17 cells (Figures 5A, B). These results suggest that AOA may impact the function of Th17 cells by inhibiting serine metabolism.

## 4 Discussion

Psoriasis is a chronic inflammatory, immune-mediated skin disease characterized by erythematous plaques covered with silvery-white scales (Lowes et al., 2014). Drugs available to treat psoriasis cause serious adverse effects. Therefore, novel effective drugs that cause fewer adverse effects are urgently needed to treat psoriasis. Through this study, we demonstrate that AOA is a potential novel treatment strategy for psoriasis.

Recent studies have revealed that keratinocytes and T cell-mediated immune responses contribute to the onset of psoriasis and its progression to chronic disease (Schlaak et al., 1994; Yuan et al., 2020). Dysfunction of T cells, including Th1, Th17, and Treg cells, and the resulting abnormal release of related cytokines that activate keratinocytes, play an important role in the onset and progression of psoriasis (Hu et al., 2021). Although psoriasis was considered a Th1-mediated skin disease in earlier studies, Th17 cells have recently been shown to play a key role in psoriatic disease (Szabo et al., 2000; Quaglino et al., 2011). In psoriasis, Th17 cells are highly activated and infiltrate the psoriatic skin lesions. The secretion of Th17-related cytokines such as IL-17A, IL-17E and IL-17F is upregulated in infiltrated Th17 cells and keratinocytes in psoriatic lesions (Martin et al., 2013). Among them, the expression of IL17A is predominant in psoriasis skin lesions, and IL-17 increases the secretion of antimicrobial peptides (*Defb4*, *Lcn2*, *S100a7*, and *S100a9*) by keratinocytes. This promotes the response of inflammatory cells and enhances the proliferation of keratinocytes (Lai et al., 2012). *Defb4* and *Lcn2* are psoriasis-specific antimicrobial peptides highly expressed in psoriatic plaques, and *Defb4* can attract Th17 cells (Kolbinger et al., 2017). *S100a7* is involved in wound healing and keratinocyte differentiation, and *S100A9* increases the production of various cytokines and chemokines in keratinocytes and is involved in keratinocyte proliferation (Hegyi et al., 2012).

According to our results, AOA reduced ear thickness and ameliorated pathological damage in mice with TPA-induced psoriasis-like skin. In addition, by confirming that AOA significantly reduced the expression of psoriasis-associated antimicrobial peptides (*Defb4*, *Lcn2*, *S100a7*, and *S100a9*), we revealed that AOA ameliorated psoriasis-related symptoms and had anti-inflammatory effects in a mouse model of psoriasis.

The exact role of Treg cells in the pathogenesis of psoriasis is largely unknown, although studies have suggested that Treg cells may ameliorate psoriasis *in vivo* (Kanda et al., 2021). Treg cells expressing FOXP3 suppress immune responses and maintain immune homeostasis. Therefore, in the treatment of psoriasis, controlling the balance between Th17 and Treg cells is key (Nussbaum et al., 2021). We have provided evidence that in a potential novel psoriasis treatment strategy, AOA can improve psoriasis by inhibiting differentiation into Th17 cells and inducing differentiation into Treg cells, thereby regulating the Th17/Treg balance. Moreover, AOA decreased IL-17A, RORC*,* and IL-22 secretion by Th17 cells, confirming that AOA is effective in the immune regulation of psoriasis.

To date, most research on psoriasis has focused on dysfunction of the immune response, although metabolite imbalance is also involved in the progression of psoriasis (Gisondi et al., 2018). Kamleh et al. found that the levels of free circulating amino acids such as arginine, alanine, glycine, serine, and threonine were altered in the plasma of patients with psoriasis (Kamleh et al., 2015). In addition, Aigar et al. demonstrated that patients with psoriasis have impaired amino acid and lipid metabolism (Ottas et al., 2017). When the immune response is activated, T cells increase the enzymes involved in serine, glycine, and 1-carbon metabolism and elevate the processing of serine into one-carbon metabolism. Therefore, serine is a highly important amino acid in the regulation of T cell activity, and reprogramming serine metabolism is key in regulating immune diseases (Ma et al., 2017).

In this study, we examined serine as an important metabolite that modulates the immune response in psoriasis, and aimed to investigate the effect of AOA on the molecular mechanisms related to serine metabolism in psoriasis. In addition, control of amino acids or inhibition of mTOR can prevent the activation of immune cells and the differentiation of keratinocytes in psoriasis (Cibrian et al., 2020). Considering these data, we sought to determine whether AMPK/mTOR is the signaling pathway involved in serine metabolism regulated by AOA regulates in psoriasis.

AMPK is an enzyme involved in cellular homeostasis and metabolic stress regulation, and phosphorylation of AMPK acts as a regulator of pathways such as AMPK/mTOR (Xiang et al., 2019). mTOR is a Ser/Thr kinase, activated mTOR is regulated by amino acid levels and controls the biosynthetic steps required for cell survival, proliferation, and cytokine release (Saxton and Sabatini, 2017). The activation of mTOR is important for the activation of Th1, Th2, and Th17 cells, whereas inhibition of mTOR induces differentiation into FOXP3^+^ Treg cells (Delgoffe et al., 2009). In keratinocytes of psoriatic skin, mTOR is upregulated by stimulation with IL-17 and IL-22, and upregulated mTOR plays an important role in the proliferation and differentiation of defective keratinocytes (Cibrian et al., 2020). The described studies can help elucidate the diagnosis and pathogenesis of psoriasis, and their use will help discover beneficial therapeutic agents for the treatment of this disease.

To elucidate the role of AOA in the treatment of psoriasis from an immunological and metabolomic standpoint, we identified a relevant mechanism in TPA-induced psoriasis mice and IL-17-stimulated human keratinocytes. AOA inhibited *Psat1, Psph*, and *Shmt1 in vivo* and significantly decreased *Phgdh, Psph, Shmt1*, and *Shmt2 in vitro*. This suggests that AOA alleviates the symptoms of psoriasis by suppressing pathway-related genes involved in serine metabolism. In addition, by confirming that AOA inhibits increased mTOR phosphorylation both *in vitro* and *in vivo*, we concluded that decreased serine by AOA is involved in the regulation of mTOR activation and suppression of mTOR phosphorylation. HaCaT cells have mainly been used for mechanistic studies, i.e., those investigating the effects of AOA on serine metabolizing enzymes and the inhibition of psoriasis-related inflammation mediated by regulating the expression of these enzymes. However, although HaCaT is a keratinocyte cell line, the results obtained with HaCaT cells may not be fully reproduced *in vivo*. Considering that we have further demonstrated, as shown in Figure 5, that AOA inhibits cytokine production and serine metabolism in Th17 cells, AOA may hold potential as a therapeutic agent in other immune diseases characterized by Th17 cell development. However, it is essential to conduct further investigations to assess its safety and efficacy.

## 5 Conclusion

AOA improved psoriasis-related symptoms by suppressing Th17-mediated cytokines, such as IL-17 and IL-22, and by reducing antimicrobial peptides in a psoriasis treatment model. In addition, AOA regulates serine by targeting and inhibiting serine metabolism-related genes and downregulating the activation of mTOR through serine metabolism control. This decreased activation of mTOR by AOA suggests that psoriasis can be alleviated by normalizing the imbalance of Th17/Treg cells. Therefore, we propose that AOA is a potential alternative treatment for relieving psoriasis.

## Data Availability

The original contributions presented in the study are included in the article/Supplementary Material, further inquiries can be directed to the corresponding authors.

## Abbreviations

AOA, aminooxy acetic acid; IL-17, interleukin-17; Th17, T-helper 17; mTOR, mammalian target of rapamycin; Treg, regulatory T cell.
