# Supplementary material for: Aminooxy acetic acid suppresses Th17-mediated psoriasis-like skin inflammation by inhibiting serine metabolism
Source: Front Pharmacol. 2023 Aug 15;14:1215861. doi: 10.3389/fphar.2023.1215861 (PMC10464615; doi:10.3389/fphar.2023.1215861)
Supplement: Supplementary file 1 [file DataSheet1.docx]

**Supplementary Information for**

**Aminooxy acetic acid suppresses Th17-mediated psoriasis-like skin inflammation by inhibiting serine metabolism**

Jong Yeong Lee, Ji-Hyun Lee, Hyo Jung Lim, Eonho Kim, Dae-Ki Kim, Jin Kyeong Choi

Corresponding author: Dae-Ki Kim and Jin Kyeong Choi

E-mail: daekim@jbnu.ac.kr (D.-K.K.); jkchoi@jbnu.ac.kr (J.K.C.)

**This file includes:**

Table S1

Figures S1

**Table S1.** Primer sequences for qPCR.

| **Gene** | **Forward** | **Reverse** |
| --- | --- | --- |
| mIL-17A | CTCAAAGCTCAGCGTGTCCAAACA | TATCAGGGTCTTCATTGCGGTGGA |
| mIL-17E | CCCCTGGAGATATGAGTTGGAC | GTCTGTAGGCTGACGCAGTG |
| mIL-17F | CAGGAAGACAGCACCATGAA | TCTTCTCCAACCTGAAGGAATTAG |
| mIL-22 | GATGAGAGAGCGCTGCTACCTGG | GAAGGACGCCACCTCCTGCATGT |
| mRorc | TGAGGCCATTCAGTATGTGG | CTTCCATTGCTCCTGCTTTC |
| mPhgdh | TGGCCTCGGCAGAATTGGAAG | TGTCATTCAGCAAGCCTGTGGT |
| mPsat1 | GATGAACATCCCATTTCGCATTGG | GCGTTATACAGAGAGGCACGAATG |
| mPsph | GAGATGGAGCTACGGACATGGAAG | CTCCTCCAGTTCTCCCAGCAGCTC |
| mShmt1 | CAGGGCTCTGTCTGATGCAC | CGTAACGCGCTCTTGTCAC |
| mShmt2 | GCGGATGTTGTTACCACC | GGGAACACAGCGAAGTTGAT |
| mGot1 | TCCATCTTTGTCCTCCATGCCTGT | AGATGCAAAGCCCTGATAGGCTGA |
| mDefb4 | CAGTCATGAGGATCCATTACCTT | AATTTGGGTAAAGGCTGCAA |
| mLcn2 | CCATCTATGAGCTACAAGAGAACAAT | TCTGATCCAGTAGCGACAGC |
| mS100a7a | CCCTGCACCAAGAGCAAC | GGACCCTTCAGGGTACAGG |
| mS100a9 | CACCCTGAGCAAGAAGGAAT | TGTCATTTATGAGGGCTTCATTT |
| mβ-actin | ACCCTAAGGCCAACCGTGAA | ATGGCGTGAGGGAGAGCATAG |
| hPhgdh | CTTACCAGTGCCTTCTCTCCAC | GCTTAGGCAGTTCCCAGCATTC |
| hPsat1 | TCAGCATCTACGTCATGGGC | TGCCAATGCGGAATGGAATA |
| hPsph | GACAGCACGGTCATCAGAGAAG | CGCTCTGTGAGAGCAGCTTTGA |
| hShmt1 | TGAACACTGCCATGTGGTGACC | CTCTTTGCCAGTCTTGGGATCC |
| hShmt2 | GCCTCATTGACTACAACCAGCTG | ATGTCTGCCAGCAGGTGTGCTT |
| hDefb4 | TGATGCCTCTTCCAGGTGTT | GCCTCCTCATGGCTTTTTGC |
| hLcn2 | CCACCTCAGACCTGATCCCA | CCCCTGGAATTGGTTGTCCTG |
| hS100a7a | ACGTGATGACAAGATTGACAAGC | GCGAGGTAATTTGTGCCCTTT |
| hS100a9 | GGTCATAGAACACATCATGGAGG | GGCCTGGCTTATGGTGGTG |
| hβ-actin | AGAGCTACGAGCTGCCTGAC | AGCACTGTGTTGGCGTACAG |


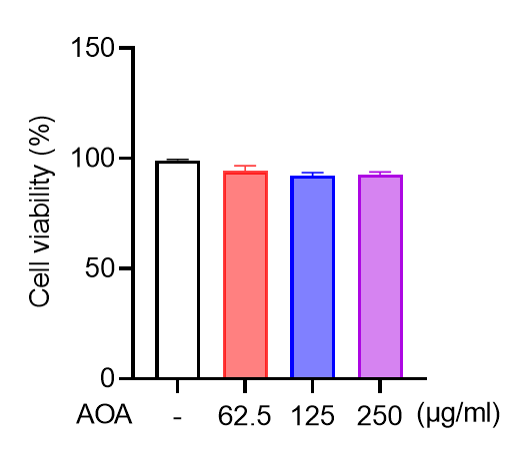


**Supplementary Fig. 1.** **Cell viability of human keratinocytes after AOA treatment.** Cytotoxicity of AOA in HaCaT cells. The data are showed as the mean ± SEM of three independent experiments. Values were analyzed by Holm-Šídák *post-hoc* test. AOA, aminooxy acetic acid; SEM, standard error of the mean; HaCaT; human keratinocytes.


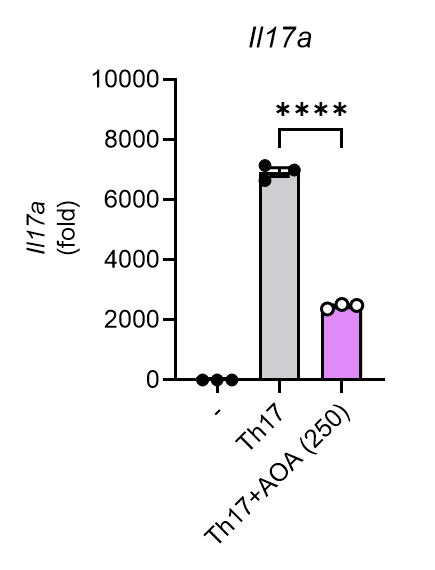


**Supplementary Fig. 2.** **AOA inhibits IL-17A production in Th17 cells.** Splenic CD4^+^CD62L^+^ naïve T cells from C57BL6/J mice were isolated and polarized under Th17 conditions with anti-CD3/28 antibodies for 5 days. The mRNA expression of IL-17A of mouse primary Th17 cells analyzed by qPCR. Values were analyzed by Holm-Šídák *post-hoc* test. *****p <* 0.0001.
